# Oral and maxillofacial surgery in patients undergoing dialysis for advanced renal disease: report of five cases

**DOI:** 10.1186/s12903-018-0634-z

**Published:** 2018-10-19

**Authors:** Yumi Mochizuki, Hiroyuki Harada, Misaki Yokokawa, Naoya Kinoshita, Kazumasa Kubota, Tomokazu Okado, Haruhisa Fukayama

**Affiliations:** 1 Department of Oral and Maxillofacial Surgery, Graduate School, Tokyo Medical and Dental University, 1-5-45 Yushima, Bunkyo-ku, Tokyo, 113-8549 Japan; 2 Department of Gerontology and Gerodontology, Gerodontology and Oral Rehabilitation, Graduate School, Tokyo Medical and Dental University, 1-5-45 Yushima, Bunkyo-ku, Tokyo, 113-8549 Japan; 3 Department of Nephrology, Graduate School, Tokyo Medical and Dental University, 1-5-45 Yushima, Bunkyo-ku, Tokyo, 113-8519 Japan; 4 Department of Anesthesiology and Clinical Physiology, Graduate School, Tokyo Medical and Dental University, 1-5-45 Yushima, Bunkyo-ku, Tokyo, 113-8549 Japan

**Keywords:** Hemodialysis, Chronic renal disease, Oral surgery, Vascularized flap reconstruction, Bone reconstruction, Surgical complication

## Abstract

**Background:**

Perioperativemanagement of hemodialysis patients involves many difficulties. High mortality rate and circulatory or respiratory complications in these patients were reported. However, in such reports, there is no concrete information of perioperative management in hemodialysis patients to prevent surgical complications and successful outcomes.

**Case presentation:**

We retrospectively reviewed the cases of 5 hemodialysis patients who underwent oral surgery under general anesthesia between January 2005 and December 2017.

Primary disease was oral squamous cell carcinoma (SCC) in 4 patients and mandibular ameloblastoma in 1 patient. Partial resection was performed in 2 cases, neck dissection in 1 case. Two cases underwent surgery including vascularized reconstruction. The patients were dialyzed the day before and after surgery for the control of fluid and electrolyte status. Patients received intraoperative and postoperative intravenous infusion of potassium-free solution at 20–40 mL/h. Erythropoiesis-stimulating agents (ESAs) were used on the day of hemodialysis during hospitalization. Nafamostat mesilate as an anticoagulant during hemodialysis were used from postoperative day (POD)1 to 7. From POD 1 to 10, cephalosporin as prophylactic antibiotics is adjusted to quarter from half the initial dose. The resuming time of oral intake was similar to that of other oral surgery patients without kidney disease. The daily intake limits of protein, salt and liquid were managed during hospitalization and no cases suffered from malnutrition. No cardiorespiratory complications occurred during the perioperative period. In a case of vascularized osteocutaneous scapular flap reconstruction, grafted scapular bone survived and scapular cutaneous flap necrotized. Necrotic tissue was debrided and split thickness skin was successfully used to cover the grafted scapular bone.

**Conclusions:**

Postoperative better result could be achieved if adequate perioperative management specific to hemodialysis patients is carried out. Vascularized flap reconstruction at oral and maxillofacial region in hemodialysis patients is beneficial treatment. Even if the first flap has wound complication secondary flap reconstruction is success and aesthetically better results could be achieved by the strict wound management and debridement.

## Background

In Japan, the number of patients on dialysis is increasing every year, reaching 314,000 at the end of 2013, three times the number 20 years earlier [1]. Given the growing number of dialysis patients, it is expected that the number of such patients undergoing surgical resection for oral disease will likewise increase.

Surgery and management of dialysis patients carries a high risk of complications, higher mortality rate, and require careful perioperative management [2–5].

There are some recent clinical studies about the head and neck surgeries in hemodialysis patients [2, 3, 5]. They suggested the mortality rate and circulatory or respiratory complications are high in these patients. However, in such reports, there is no concrete information of perioperative management in hemodialysis patients to prevent surgical complications and successful outcomes.

## Objective

We retrospectively reviewed the cases of 5758 patients who underwent oral surgery under general anesthesia at our department between January 2005 and December 2017. Among these cases, 5 patients were receiving hemodialysis. In this study, we evaluated the perioperative management and outcomes of oral surgeries, including vascularized bone or flap reconstructions, in patients undergoing hemodialysis. We discuss and suggest the specific and successful management of oral surgeries including vascularized bone or flap reconstructions in dialysis patients.

## Case presentation

### Patients’ summary were showed in Table 1

#### Case 1

A 28 year-old man was referred to our department for the treatment of tongue carcinoma. The etiology of dialysis-dependent end-stage kidney disease was Alport’s syndrome and the duration of hemodialysis treatment was 7 years and 9 months. His history included hypertension and anemia. No metastatic lymph node was palpable in the cervical region and the clinical diagnosis was tongue cancer. Partial glossectomy was performed (surgical time; 47 min (min), intraoperative bleeding loss volume; 63 ml). Intravenous second-generation cephalosporin 0.5 g was administrated just before the surgery. Intraoperative intravenous infusion volume of potassium-free solution was 200 mL (mean infusion speed at 20–40 mL/h). Routine hemodialysis was scheduled for 2 days before and after surgery, and then 3 times a week. We discussed patients’ conditions and perioperative dialysis management with nephrologists once a week during hospitalization. Erythropoiesis-stimulating agents (ESAs) were used on the day of hemodialysis during hospitalization. Nafamostat mesilate as an anticoagulant during hemodialysis was used from POD 1 to 7. From POD 1 to 5, the second-generation cephalosporin (0.5 g once daily intravenously) and the third-generation cephalosporin (0.2 g once daily per mouth) during POD 6 to 10 were administered. The healing process was uneventful and oral intake was resumed on POD 5. Daily limits of protein intake, salt intake, and liquid intake were 70 g, 7 g and 500 mL, respectively. The pathological diagnosis of surgical specimen was squamous cell carcinoma (SCC). No adjuvant therapy was performed. The patient was free of the disease 13 years after surgery.

**Table 1:** Patients’ background and treatments summary

Case No.	Age	Sex	Diagnosis Treatment	Renal disease General medical history	The duration of dialysis treatment	Surgical time (hour:h, minute:min)	Fluid volume (ml)	Bleeding volume (ml)	The start day of oral intake after surgery (post operative day)	Protein intake (g)	Salt intake (g)	Daily limit of liquid intake (ml)
1	28	M	Tongue cancer T2 N0 Partial glossectomy	Alport’s syndrome Hypertension Anemia	7y 9m	47 min	200	63	5	70	7	500
2	37	M	Tongue cancerT3N2C Tracheotomy +bi-lateral neck dissection +subtotal glossectomy + vascularized abdominal flap reconstruction	Chronic kidney failure Hypertension Lacunar infarction Hepatitis C Secondary hyperparathyroidism Anemia	9y 4 m	10 h 36 min	814	514	21	50	5	1500
3	55	M	Lower gingival cancerT2 N0 Marginal mandibulectomy	Chronic glomerulonephritis Anemia Peptic ulcer	17y 6 m	2 h 13 min	250	770	7	70	7	1000
4	72	M	Subsequently cervical lymph node metastasis after brachy therapy of buccal SCC Neck dissection (Level I-IV)	Chronic kidney failure after kidney cancer surgery Hypertension Secondary hyperparathyroidism Anemia	1y10m	4 h 49 min	313	131	0^a^	70	7	500
5	65	M	Mandibular ameloblastoma 1.Segmental mandibulectomy +plate reconstruction 2.Plate removal +vascularized osteocutaneous scapular flap reconstruction 3.Spilt thickness skin graft	Diabetic nephropathy Hypertension Diabetes Diabetic retinopathy Cerebral infarction Secondary hyperparathyroidism Anemia	2y 8 m	5 h 9 min 9 h 42 min 1 h 22 min	166 1075 23	97 209 6	13 14 0^a^	60 60 60	6 6 6	800 800 800
Median						4 h 36 min	250	96	10	65	6.5	800

^a^Start at operative day

*y* Year, *m* Month, *h* Hour, *min* Minute

#### Case 2

**Fig. 1:**
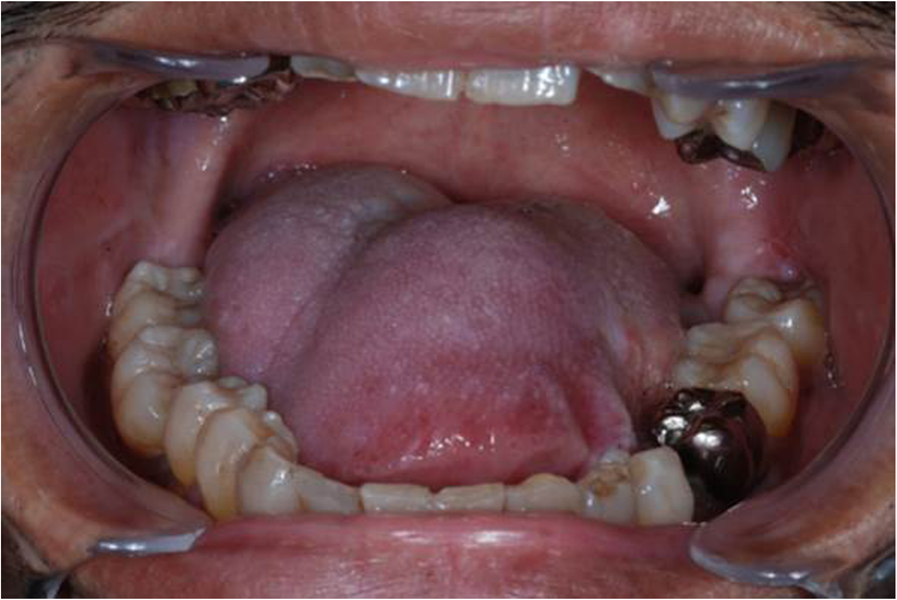
Oral photograph at first visit. Oral photograph showed 5.0 × 4.0 × 2.6-cm tumor on the left side of the tongue

**Fig. 2:**
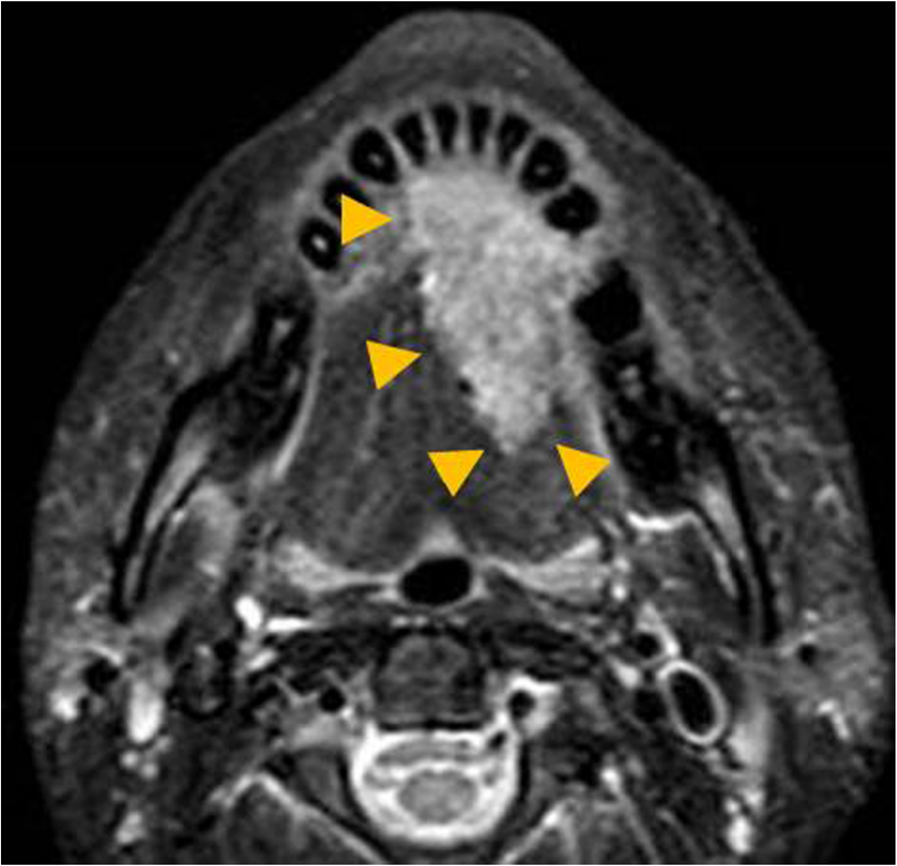
MRI before surgery. T2 weighted MRI showed that the tumor occupied beyond the half of the tongue

**Fig. 3:**
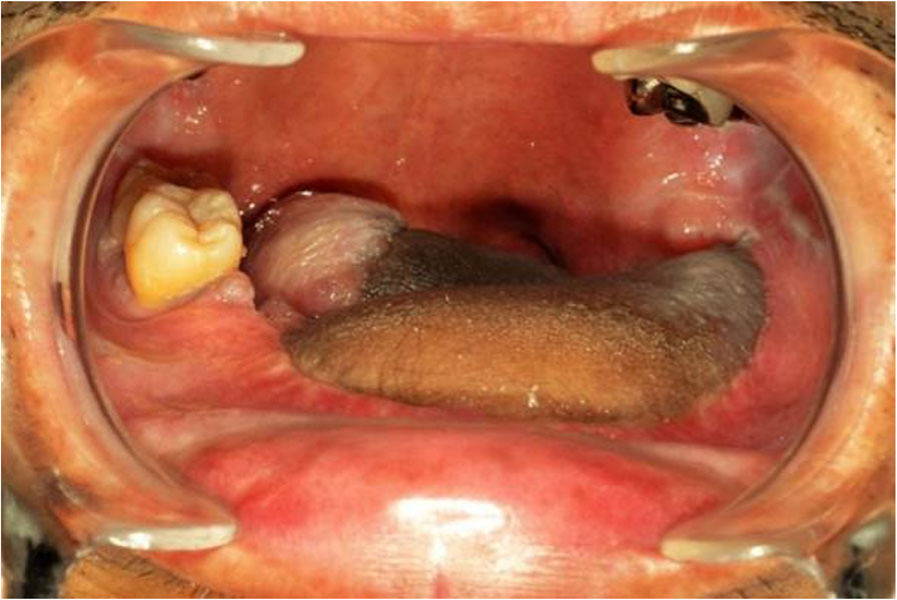
Oral photograph after flap reconstruction. Oral photograph after subtotal glossectomy and abdominal vascularized flap reconstruction

A 37-year-old man, initially treated with partial glossectomy for tongue SCC, was referred to our department for recurrence. The cause of dialysis-dependent disease was chronic kidney failure, and the duration of dialysis treatment was9 years and 4 months. His medical histories were hypertension, anemia, secondary hyperparathyroidism, lacunar infarction, and hepatitis C. Oral examination revealed an endophytic tumor with mucosal ulceration on the left side of the tongue (Fig. 1). Preoperative magnetic resonance imaging (MRI) demonstrated a tumor measuring 5.0 × 4.0 × 2.6 cm (Fig. 2) and bilateral cervical lymph node metastasis. Tracheotomy, bilateral neck dissection, (ipsilateral: Level I-IV, contra lateral: Level I-III) and subtotal glossectomy were performed with abdominal vascularized flap reconstruction (surgical time; 10 h (h) 36 min, intraoperative bleeding loss volume; 514 mL). Intravenous second-generation cephalosporin 1 g just before the surgery was used and intraoperative intravenous infusion volume of potassium-free solution was 814 mL (mean infusion speed at 20–40 mL/h). Routine hemodialysis was scheduled for the day before and after surgery, and then 3 times a week. We discussed patients’ conditions and perioperative dialysis management with nephrologists once a week during hospitalization. ESA was used on the day of hemodialysis during hospitalization. Nafamostat mesilate was used from POD 1 to 7. From POD 1 to 5, the second-generation cephalosporin (1 g once daily intravenously) and the third-generation cephalosporin (0.2 g once daily per mouth) during POD 6 to 10 were administered. The healing process was uneventful and oral intake was restarted on POD 21. Daily limits of protein intake, salt intake, and liquid intake were 50 g, 5 g and 1500 mL, respectively. The pathological diagnosis of surgical specimen was SCC and four cervical lymph node metastasis (level II and level III at ipsilateral side, level II and level III at contra lateral side). Postoperative oral photograph is shown in Fig. 3. Pain control was achieved by using pentazocine hydrochloride and oxycodone hydrochloride hydrate. Adjuvant radiation therapy (50 Gy) was administered to the primary oral lesion and neck lesions bilaterally. Four months after the surgery, the primary tumor recurred, and he died 9 months later.

#### Case 3

A 55 year-old man visited to our department for the treatment of lower gingival carcinoma. The etiology of dialysis-dependent end-stage kidney disease was Chronic glomerulonephritis and the duration of hemodialysis treatment was17 years and 6 months. Peptic ulcer and anemia had been treated. No metastatic lymph node was palpable in the cervical region and the clinical diagnosis was lower gingival cancer. Marginal mandibulectomy was performed (surgical time; 2 h 13 min). At the day before surgery red blood cells were transfused because of Hb value of 7.0 g/dL and a preoperative Ht value of 22.0% caused by gastrointestinal bleeding. We administrated intravenous second-generation cephalosporin 0.5 g just before the surgery. Intraoperative intravenous infusion volume of potassium-free solution was 250 mL (mean infusion speed at 20–40 mL/h). Routine hemodialysis was scheduled for the day before and after surgery, and then 3 times a week. We discussed assessment of patients’ conditions and perioperative dialysis management with nephrologists once a week during hospitalization. As POD 1 day after surgery, Hb and Ht levels were still low (Hb 6.5 g/dL, Ht 20.5%), and red blood cells were transfused per each day at POD on the 3, 6, and 8 days after surgery. By POD the 13 days after surgery, Hb and Ht levels improved (Hb 9.9 g/dL, Ht 31.0%). ESA was used on the day of hemodialysis during hospitalization. Nafamostat mesilate was used from POD 1 to 7. From POD 1 to 5, the second-generation cephalosporin (0.5 g once daily intravenously) and the third-generation cephalosporin (0.2 g once daily per mouth) during POD 6 to 10 were administered. The healing process was uneventful and oral intake was resumed on POD7. Daily limits of protein intake, salt intake, and liquid intake were 70 g, 7 g and 1000 mL, respectively. The pathological diagnosis of surgical specimen was SCC. The margin of the surgical specimen was free of tumor. No adjuvant therapy was performed. The patient was free of the disease 11 years after surgery.

#### Case 4

A 72 year-old man was referred to our department for the treatment of subsequent cervical lymph node metastasis 4 months after brachy therapy of buccal SCC. For primary lesion, he received brachytherapy (first doze; 84Gy, second doze for tumor remaining; 83.97Gy) and external irradiation (30Gy). The etiology of dialysis-dependent end-stage kidney disease was chronic kidney failure after kidney cancer surgery and the duration of hemodialysis treatment was 1 year and 10 months. His history included hypertension, secondary hyperparathyroidism and anemia. Neck dissection (Level I-IV) was performed (surgical time; 4 h 49 min, intraoperative bleeding loss volume; 131 mL). Intravenous first-generation cephalosporin 0.5 g just before the surgery was used. Intraoperative intravenous infusion volume of potassium-free solution was 313 mL (mean infusion speed at 20-40 mL/h). Routine hemodialysis was scheduled for the day before and after surgery, and then 3 times a week. We discussed patients’ conditions with nephrologists once a week during hospitalization. ESA was used on the day of hemodialysis during hospitalization. Nafamostat mesilate as an anticoagulant during hemodialysis were used from postoperative day (POD) 1 to 7. From POD 1 to 5, first-generation cephalosporins (0.5 g once daily intravenously). Oral intake was restarted on the operative day. Daily limits of protein intake, salt intake, and liquid intake were 70 g, 7 g and 500 mL, respectively. The pathological diagnosis of surgical specimen was one cervical lymph node metastasis at Level II. Postoperative adjuvant chemotherapy was not administrated. Because of the general weakness caused by rapid progress of osteoradionecrosis of the mandible and disability of oral intake he could not come to our department and transferred to another hospital 1 year and 3 months after neck dissection.

#### Case 5

**Fig. 4:**
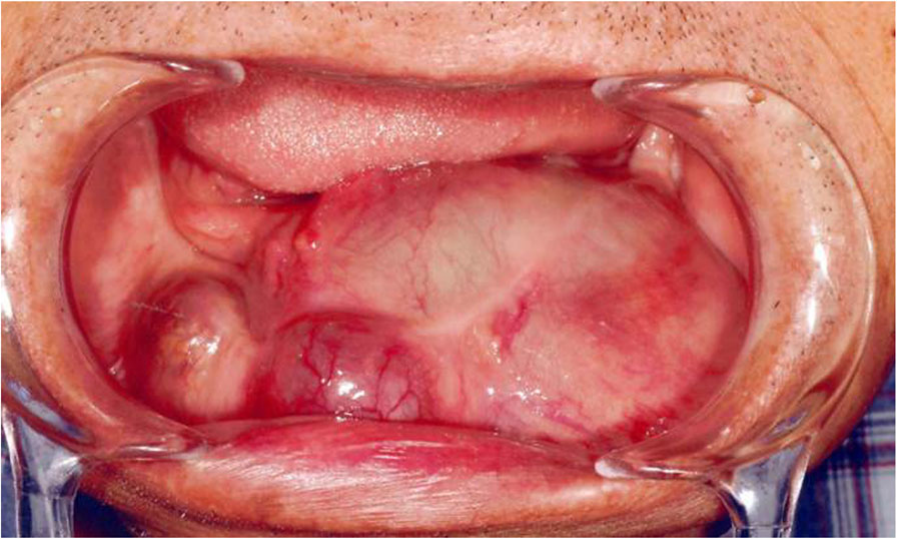
Oral photograph at first visit. Multiple swelling mass covered with normal mucosa was observed from the right lower molar area to the left lower molar area

**Fig. 5:**
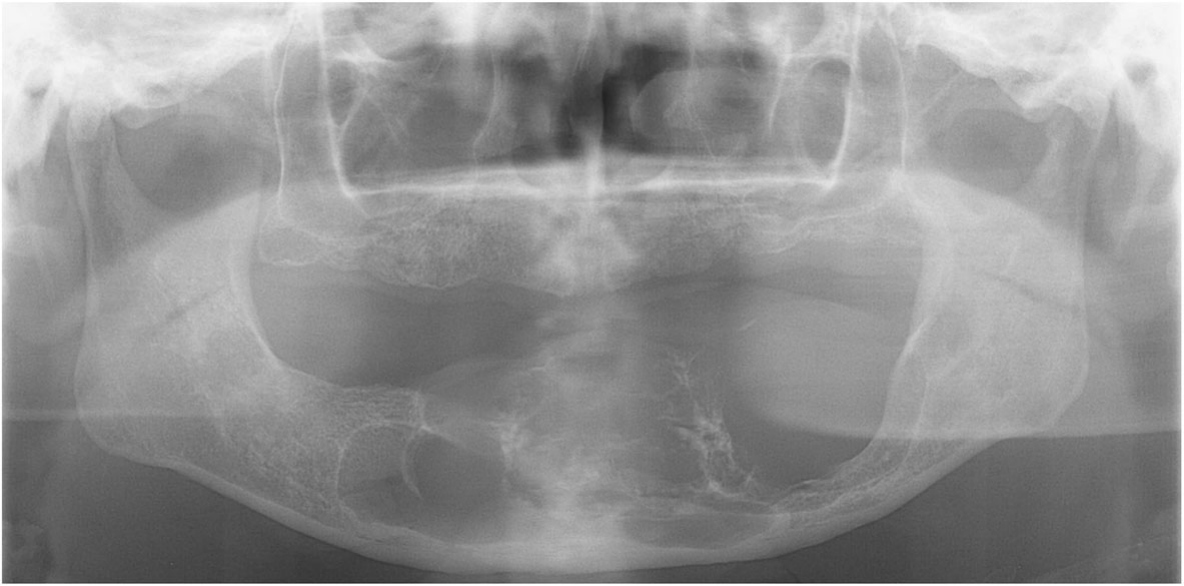
Panoramic radiograph at first visit. Panoramic radiograph showed a well-defined radiolucent multilocular mass in the bilateral mandible

**Fig. 6:**
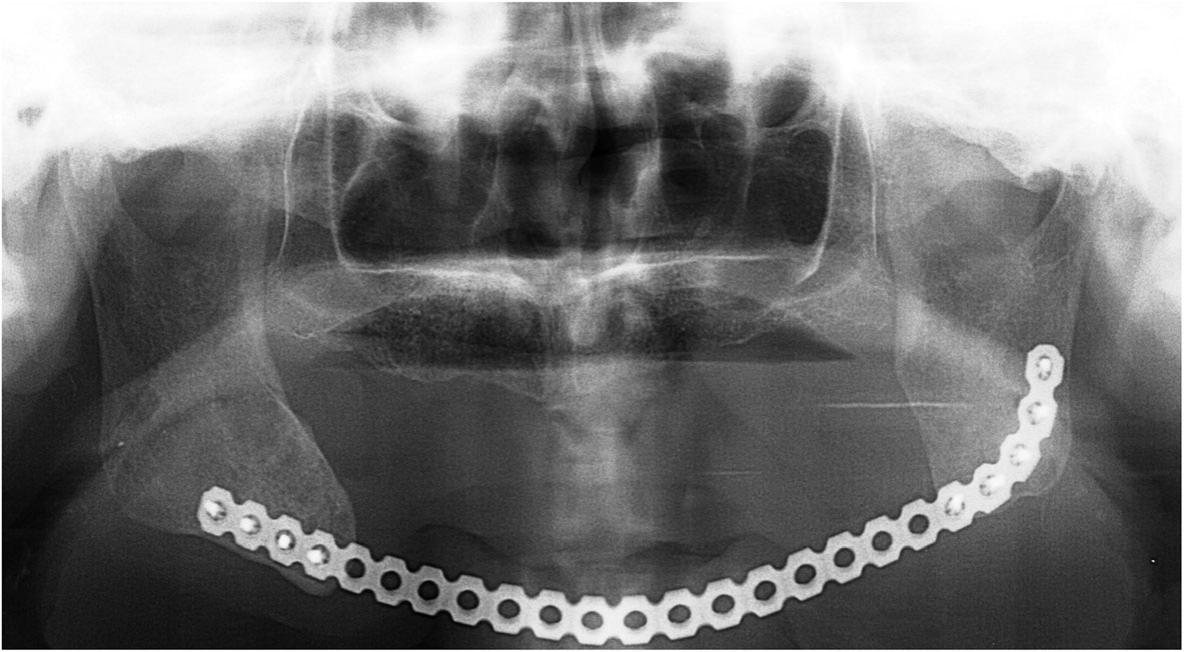
Panoramic radiograph after plate bone reconstruction. The mandibular defect after segmental mandibulectomy was reconstructed by the titanium plate

A 65-year-old man on hemodialysis was referred to our department for a mandibular tumor. The duration of hemodialysis treatment was2 years and 8 months. His medical history included diabetes, hypertension, cerebral infarction, diabetic retinopathy, and secondary hyperparathyroidism. On oral examination, a huge mass was observed in the right lower molar area extending to the left lower molar area (Fig. 4). Panoramic radiography showed a well-defined radiolucent multilocular mass in the mandible (Fig. 5). Segmental mandibulectomy and plate reconstruction were performed (surgical time; 5 h 9 min, intraoperative bleeding loss volume; 97 mL) (Fig. 6). Intravenous second-generation cephalosporin (1 g) was administrated just before the surgery. Intraoperative intravenous infusion volume of potassium-free solution was 166 mL (mean infusion speed at 20–40 mL/h) and included glucose-insulin-potassium (GIK) therapy. Routine hemodialysis was scheduled for the day before and after surgery, and then 3 times a week. We discussed patients’ conditions and perioperative dialysis management with nephrologists once a week during hospitalization. ESA was used on the day of hemodialysis during hospitalization. Nafamostat mesilate as an anticoagulant during hemodialysis were used from postoperative day (POD) 1 to 7. From POD 1 to 5, the second-generation cephalosporin (1 g once daily intravenously) and the third-generation cephalosporin (0.1 g once daily per mouth) during POD 6 to 10 were administered. Wound healing was uneventful and oral intake was restarted on POD 13. Daily limits of protein intake, salt intake, and liquid intake were 60 g, 6 g and 800 mL, respectively. The pathological diagnosis of surgical specimen was ameloblastoma. No recurrence was observed during the follow-up period.

**Fig. 7:**
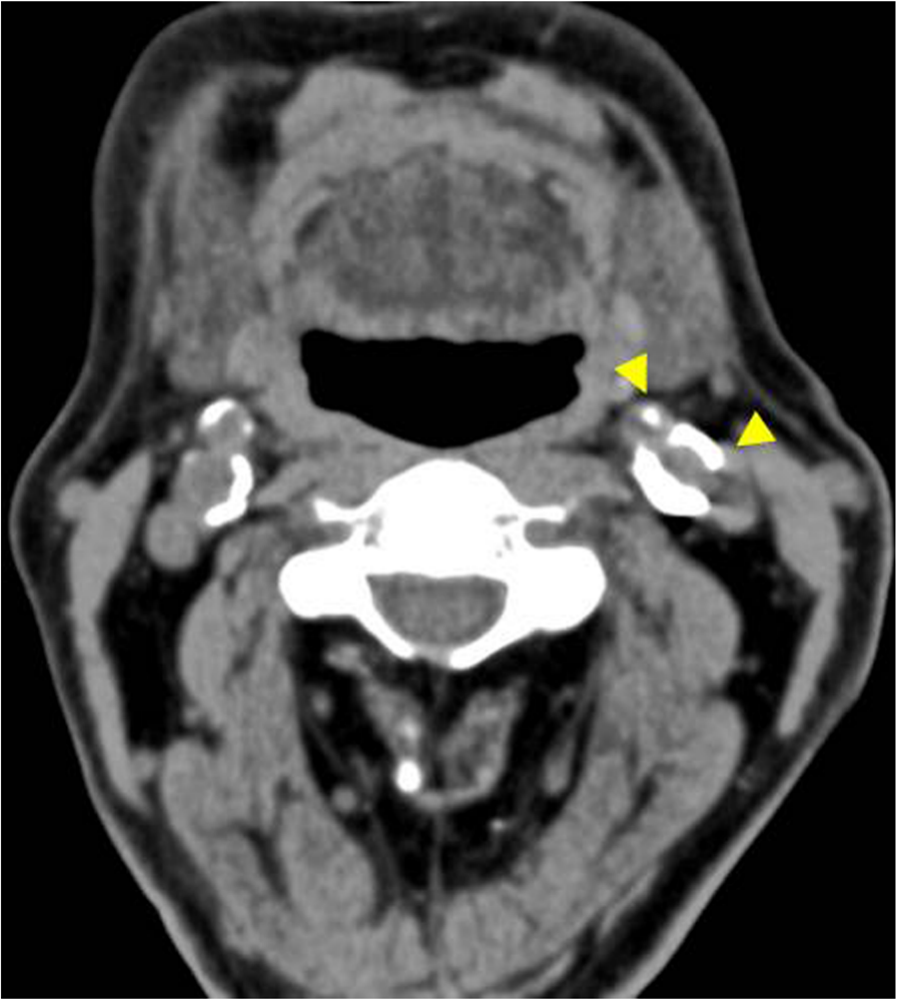
CT scan image (Axial view). In preoperative CT, vascular calcification of carotid arteries on both sides was observed (yellow arrows)

**Fig. 8:**
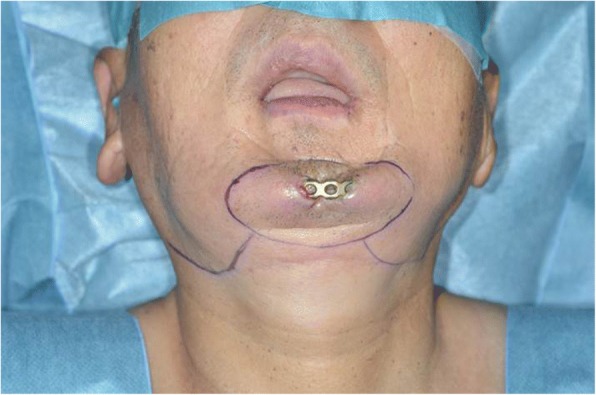
Facial photograph during surgery. The mentum skin around the plate exposure was resected

**Fig. 9:**
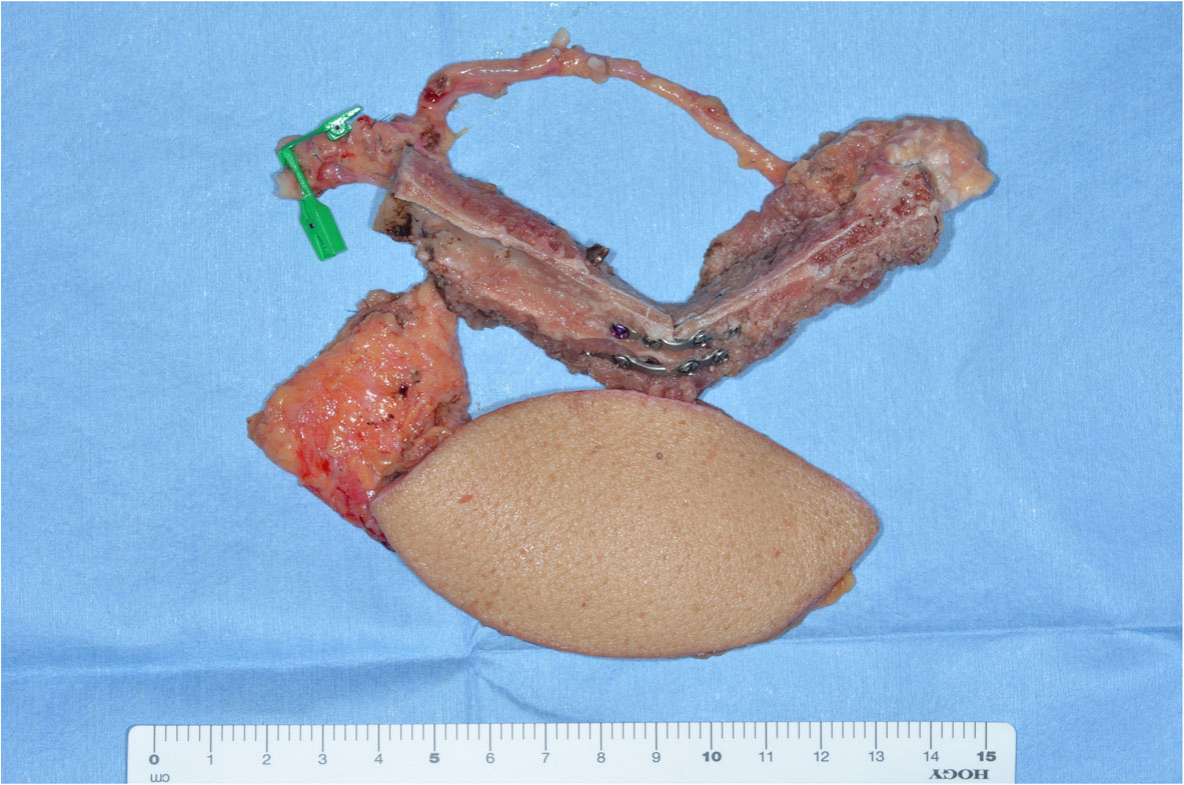
Photograph of scapular bone flap and cutaneous flap. Scapular bone was bended to fit the morphology of the mandible and fixed. Scapular cutaneous flap reconstructed the resected facial skin

**Fig. 10:**
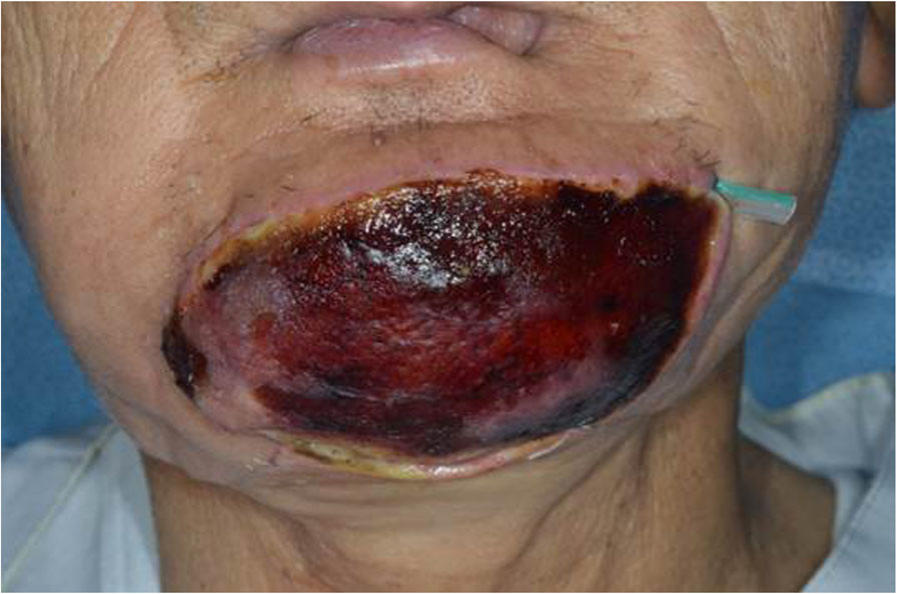
Facial photograph of wound complication. Subcutaneous suggillation emerged above the scapular flap. Pus discharged from the suture line of scapular cutaneous flap and drain tube was held. Flap totally necrotized

**Fig. 11:**
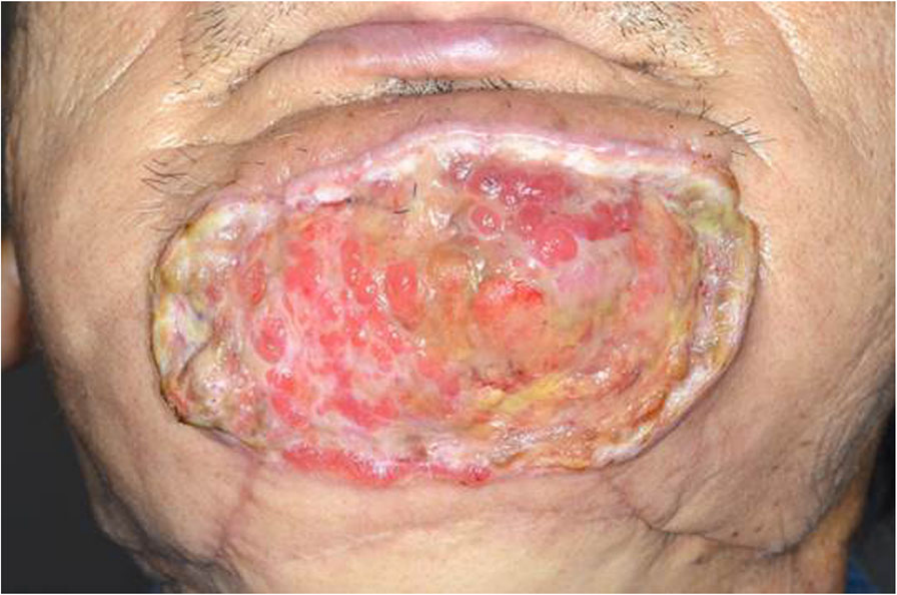
Facial photo graphs after necrotic tissue debridement. Granulation tissue over the grafted scapular bone was observed after debridement of necrotic tissue

**Fig. 12:**
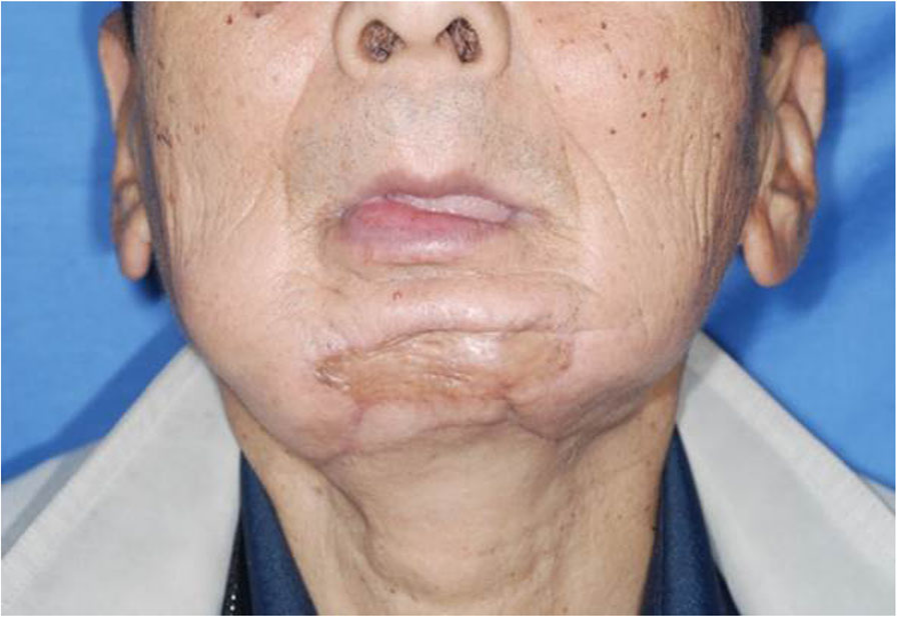
Postoperative facial photograph. Facial photograph at postoperative 3 months after split thickness skin graft

**Fig. 13:**
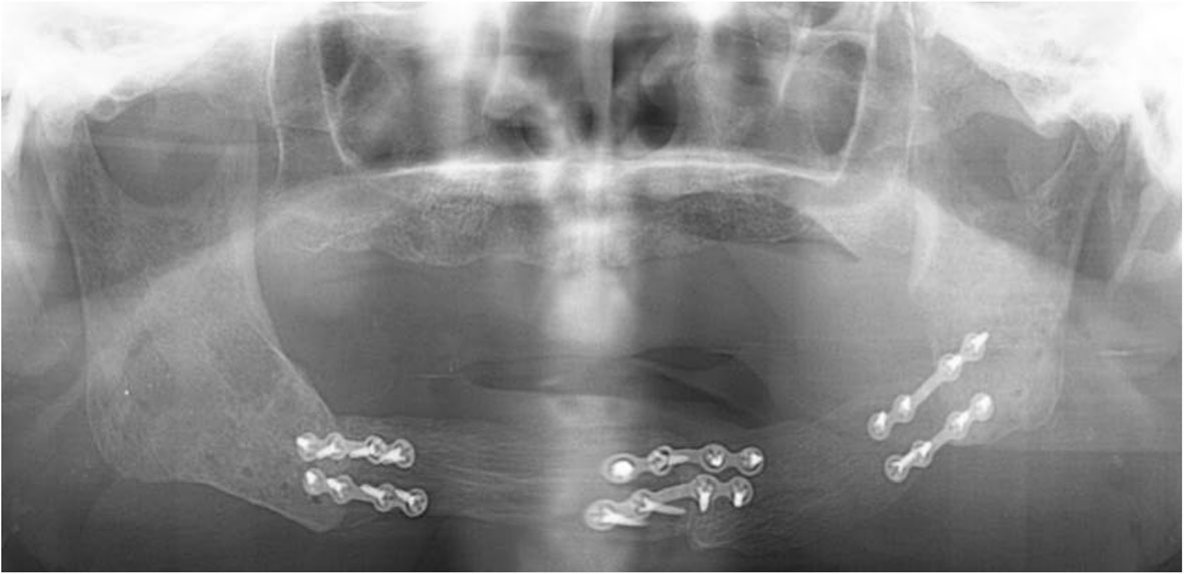
Panoramic radiograph after scapular bone reconstruction. Panoramic radiograph showed that the mandible was reconstructed with scapular bone after the plate removal

At 6 years after the first surgery, exposure of the plate was noted. We provided treatment options of plate removal alone, or reconstruction of an autologous bone graft. The patient chose the autologous bone graft. Diabetic control was poor (HbA_1C_ 9.2%) and adequate blood glucose control was ensured with intensive insulin therapy. Preoperative computed tomography (CT) showed vascular calcification of the carotid arteries on both sides (Fig. 7). However, the Doppler signals of the facial and superior thyroid artery to be anastomosed were observed. For the second surgery, plate removal and vascularized osteocutaneous scapular flap reconstruction were performed (surgical time; 9 h 42 min, intraoperative bleeding loss volume; 209 mL) (Figs. 8 and 9). Intravenous second-generation cephalosporin (1 g) was administrated just before the surgery. Intraoperative intravenous infusion volume of potassium-free solution was 1075 mL (mean infusion speed at 20–40 mL/h) and included GIK therapy. The schedule of routine hemodialysis, discussion with nephrologists, drug regimen of ESA, Nanafamostat mesilate and the antibiotics were the same as the first surgery. Red blood cells were transfused because Hb and Ht levels gradually decreased to 6.5 g/dL and 20.2%, respectively, on POD 7. Bone scintigraphy of radiolabeled ^99m^Tc-methylene-diphosphonate imagin*g* showed viability of the vascularized bone graft (POD 5), however, the scapular cutaneous flap began to necrosis on POD 9. Wound infection with methicillin-resistant *Staphylococcus aureus* (MRSA) was noted on culture. Vancomycin (0.5 g) was interveneously administered on the day of hemodialysis. Oral intake was uneventfully restarted on POD 14. The daily intake limits of protein, salt and liquid were the same as the first surgery. The scapular cutaneous flap underwent necrosis completely by POD 16 (Fig. 10). On POD 37 we performed the necrotic tissue debridement and found the formation of granulation tissue on the surface of scapular bone (Fig. 11). We performed the necrotic tissue debridement and split thickness skin graft on the scapular bone (surgical time; 1 h 22 min, intraoperative bleeding loss volume; 23 mL). Intravenous second-generation cephalosporin (1 g) was administrated just before the surgery. Intraoperative intravenous infusion volume of potassium-free solution was 23 mL (mean infusion speed at 20–40 mL/h). The schedule of routine hemodialysis, discussion with nephrologists, drug regimen of ESA, Nanafamostat mesilate and the antibiotics were the same as the first surgery and second surgery. Oral intake was restarted on the operative day. The daily intake limits of protein, salt and liquid were the same as the first surgery and second surgery. The healing process was uneventful (Figs. 12 and 13). The patient was free of the disease 3 years after surgery.

## Discussion

### Perioperative management

In all our cases, there were no cardiorespiratory complications during the perioperative period. The dialysis schedule, the volume of intravenous fluid infusion and the liquid limitation volume of liquid intake were considered appropriate.

In hemodialysis patients, pulmonary edema and poorly-controlled hypertension are easily induced by volume overload. Furthermore, vascular abnormalities such as atherosclerotic plaques, vascular calcification in hemodialysis patients cause delayed response to fluid volume changes, which could result in irreversible complications such as colonic necrosis and brain infarction [6]. Thus, circulating fluid volume and arterial pressure during the perioperative period should be strictly monitored [4, 7].

Patients are typically scheduled to be dialyzed the day before surgery and re-started the day after surgery, for control of fluids, minerals, and electrolyte metabolism [4]. During the intraoperative period, anesthesiologists and oral surgeons should consider the possibility of hypovolemia and profound hypotension induced by intraoperative blood loss and general anesthesia [5].

Patients with chronic kidney disease have disorders of mineral and electrolyte metabolism [4, 7]. It is necessary to pay attention to hyperkalemia in the perioperative period. In diabetic nephropathy, potassium and blood glucose management by GIK therapy is also considered useful [8].

Patients on hemodialysis have increased bleeding tendency because of platelet dysfunction and the heparinization employed in routine hemodialysis [9]. It is reported that using ultra-short acting nafamostat mesilate for 1 week postoperatively is effective in preventing postoperative bleeding and hematoma [9].

Management of patients with chronic kidney disease involves addressing anemia from impaired erythropoietin secretion [9]. Brattich et al. reported that patients with chronically low Ht and Hb (Ht < 30%, Hb < 8 g/dL) had relatively higher mortality rates among dialysis patients [10]. Therapy with iron supplementation (such as intravenous iron) and ESAs is effective for postoperative anemia [4]. However, there are no optimal standard Hb and Ht levels in hemodialysis patients during the perioperative period [4]. In our case series, all patients were administered ESAs, and blood transfusion was performed in 2 cases with Ht < 22% and Hb < 7.0 g/dL.

Hemodialysis patients are at increased risk of infection due to systemic immune dysfunction [11] and impairment of the wound healing process [2, 12]. In addition to these risks, many oral, head and neck surgical wounds are generally considered clean-contaminated [13]. For these reasons, perioperative antibiotic prophylaxis is recommended for oral surgery in hemodialysis patients [14]. The dosage of prophylactic antibiotics is adjusted to quarter from half the initial dose in patients with chronic kidney disease [15]. The administration schedules considering the dialysis day should be discussed among the patient’s nephrologists [15].

In our cases, there were no severe systemic infections or drug toxicity with the administrated dosage of prophylactic antibiotics.

### Oral intake

Hemodialysis patients are at increased risk of delayed wound healing, wound dehiscence, and malnutrition [2, 4, 12]. We strictly maintained close apposition of the intraoral wound margins, prevented intraoral wound dehiscence and surgical dead space. In our cases, the time course of intraoral wound healing, wound management and the resuming time of oral intake were similar to that of other oral surgery patients without kidney disease and no cases suffered from malnutrition.

Our results suggested that by close management of intraoral wound margins and dead space oral intake was not prolonged after oral surgery in hemodialysis patients.

### Adaptation of vascularized free flap reconstruction

Moran S. L. suggested that vascularized free tissue transfer should not be considered as a contraindication in hemodialysis patients [2]. However, there is a high incidence of pre-existing endothelial dysfunction and microvascular perfusion abnormalities [16] and peripheral arterial disease in patients undergoing hemodialysis, especially diabetic angiopathy in diabetes mellitus patients [17, 18]. In general, arteriosclerosis or vascular calcification often causes thrombosis after vascular anastomosis [19]. Thus, such risks of vascular anastomosis in hemodialysis patients should be considered [2, 5]. As far as we detected, there is one latest study of vascular reconstruction and free flap transfer in Gangrenous lesions of the foot or lower leg due to severe diabetic arterial disease [20]. On the other hand, in the field of head and neck reconstruction, there are only few reports of vascularized bone flap reconstructions in chronic kidney patients with diabetic nephropathy [2].

In our case 5 with diabetic nephropathy, grafted scapular bone survived and scapular cutaneous flap necrotized. However, split thickness skin was successfully grafted on viable scapular bone after complete debridement of necrotic scapular cutaneous flap and achieved aesthetically better result. Focusing on our results and previous report [2], aggressive wound debridement and eradication of infection before secondary flap transfer could improve flap survival and aesthetically better results could be achieved even at the secondary salvage oral and maxillofacial surgery in hemodialysis patients.

In patients with chronic kidney disease renal osteodystrophy has been reported [4]. In consideration of our results of successful scapular bone union, vascularized bone flap can be used in hemodialysis patients with a medical history of secondary hyperparathyroidism.

Our presented case was the rare case on vascularized scapular osteocutanous flap reconstruction for the mandible defect in patients on hemodialysis. Further clinical studies for vascularized flap reconstruction at oral and maxillofacial region in hemodialysis patients is needed.

## Conclusions

We presented 5 case series and beneficial information of perioperative successful management for the oral surgery in hemodialysis patients. We concluded postoperative better result could be achieved if adequate perioperative management specific to hemodialysis patients is carried out. We also presented the vascularized scapular osteocutaneous flap reconstruction in hemodialysis patient with diabetic nephropathy and secondary hyperparathyroidism. We insisted that even if the first flap has wound complication secondary flap reconstruction is success and aesthetically better results could be achieved by the strict wound management and debridement.

### Consent

Written informed consent was obtained from the parents for publication of this case report and any accompanying images. A copy of the written consent is available for review.

## Abbreviations

CT: Computed tomography
ESA: Erythropoiesis-stimulating agent
GIK: Glucose-insulin-potassium
HB: Hemoglobin
Ht: Hematocrit
MRI: Magnetic resonance imaging
MRSA: Methicillin-resistant *Staphylococcus aureus*
POD: Postoperative day
SCC: Squamous cell carcinoma
